# Supplementary material for: Functional Characterization of the MYO6 Variant p.E60Q in Non-Syndromic Hearing Loss Patients
Source: Int J Mol Sci. 2022 Mar 21;23(6):3369. doi: 10.3390/ijms23063369 (PMC8949016; doi:10.3390/ijms23063369)

Figure S1. Effect on zebrafish survival and hatching rates after Human mRNA *MYO6* co-injections with morpholino targeting zebrafish *myo6a* ATG translation blocking site:

A. Injection of MO_ATG, Human $MYO6^{WT}$ and $MYO6^{p.E60Q}$ variant had no effect on zebrafish survival rate at 24 hours post fertilization (hpf), p-value < 0.54. **B.** Injection of MO_ATG, $MYO6^{WT}$ and $MYO6^{p.E60Q}$ variant had negative effect on hatching rate at 72 hpf, p-value < 0.00001. Total number of experiments= 4, Number of embryos injected were ~50-100 embryos for each examined group. Statistical analysis performed using Chi-square comparison.

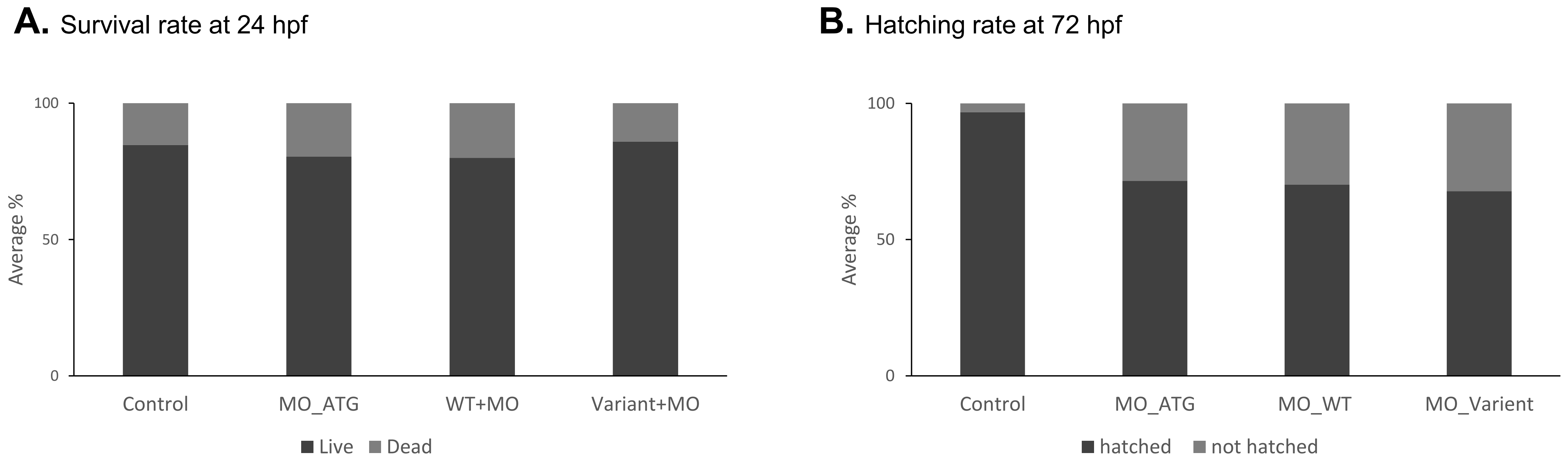

Supplement: Supplementary file 1 [file ijms-23-03369-s001.zip › ijms-1617460-supplementary.pdf]
